# Guidance for Genuine Collaboration: Insights from Academic, Tribal, and Community Partner Interviews on a New Research Partnership

**DOI:** 10.3390/ijerph16245132

**Published:** 2019-12-16

**Authors:** Orly Stampfer, Gillian Mittelstaedt, Victoria Breckwich Vásquez, Catherine J. Karr

**Affiliations:** 1 Department of Environmental and Occupational Health Sciences, University of Washington, Seattle, WA 98105, USA; 2 Tribal Healthy Homes Network, Issaquah, WA 98029, USA; gmittelstaedt@thhnw.org; 3 Doctor of Public Health Leadership Student, University of Illinois at Chicago, Chicago, IL 60607, USA; 4 Sea Mar Community Health Centers, Seattle, WA 98108, USA; victoriabreckwich@seamarchc.org; 5 Department of Pediatrics, University of Washington, Seattle, WA 98105, USA; ckarr@uw.edu; 6 Northwest Pediatric Environmental Health Specialty Unit, University of Washington, Seattle, WA 98105, USA

**Keywords:** community-engaged research, community-based participatory research, community–academic partnership, tribal–academic partnership, capacity building, collaboration

## Abstract

As community engaged research (CEnR) increases in popularity and recognition, specific guidance on partnership approaches that are more likely to lead to community benefits is needed. Here, we describe a qualitative interview study aimed at better understanding community and academic perspectives on elements of genuine collaboration within a project’s new community–academic partnership. This partnership involved a large, public, urban university, a tribal nation government program, a small, rural, community-based university, and a local high school working together to develop CEnR on air quality. Interview questions were formulated from a literature review examining the relationships between trust, cultural relevance, and community involvement in research with partnership processes, roles, and strengths. Twelve semi-structured interviews were conducted with individuals from the community–academic partnership: six University of Washington research team members and six community partners. Guidance for an authentic collaborative partnership supported by interview analyses includes incorporating elements of partnership and project sustainability from the earliest phases and throughout; promoting funding mechanism responsiveness to relationship building and community partner involvement in budget decision-making; acknowledging community strengths, knowledge, and expertise and applying them; establishing roles that reflect community partner capacity building goals; and recognizing community diversity and dynamics to promote representation.

## 1. Introduction

As community engaged research (CEnR) increases in popularity and recognition, specific guidance on partnership approaches that are more likely to lead to community benefits is needed [1]. Ahmed and Palermo define CEnR as: “a process of inclusive participation that supports mutual respect of values, strategies, and actions for authentic partnership of people affiliated with or self-identified by geographic proximity, special interest, or similar situations to address issues affecting the well-being of the community of focus” [2]. CEnR methods were highlighted for their role specifically in advancing environmental health [3]. Research centered on CEnR principles in rural areas has been successful in engaging communities to explore health-related topics [4,5]. CEnR has been shown to be especially successful among Native communities because it facilitates the application of tribal expertise and knowledge in research, aligns with some existing tribal research protocols, and increases research capacity among tribes and community partners [6,7,8]. While there are many benefits to CEnR, inauthentic partnerships can be exploitative and further marginalize and oppress communities [9,10]. Therefore, it is important to have additional guidance in establishing meaningful partnerships, informed by both academic and community partners.

Trust, equitable and culturally informed processes, and community involvement in research have been previously identified as components underlying successful CEnR practice [11,12,13,14]. Here we describe a qualitative interview study aimed at better understanding community and academic perspectives on elements of genuine collaboration within the project’s new community–academic partnership.

Few research groups employing CEnR, community-based participatory research, community–academic partnerships, tribal–academic partnerships, and other forms of collaborative and participatory research have evaluated their own practices for success. Methodologies for evaluation include review through the Good Practice of Community Campus Partnerships framework [9]; review through the Contact, Initiation, Acceptance, Success, and Continuation Evaluation Framework [15]; analysis of meeting minutes and a group meeting [10]; analysis of program documentation, partner surveys, and meeting minutes [16]; and post-meeting evaluations [17]. The National Institute of Environmental Health Sciences’ Partnerships for Environmental Public Health released an evaluation metrics manual to measure success in partnerships; the manual was based on academic and community partner input [18].

Generally, research process evaluations occur at the end of a project or between major phases. This paper shares perspectives of academic and community partners at the beginning of a research collaboration. While this analysis includes some retrospective evaluation, much of it is hypothetical, reflecting how partners imagine the partnership could be ideally. This work reflects perspectives on CEnR that are less influenced by completed partnership processes. Interestingly, there is much overlap in themes and implications from the perspectives analyzed here, from the beginning of the partnership, with perspectives analyzed in other papers, generally from the end of at least the first project in a partnership.

### Background on the CEnR partnership

Rural lower Yakima Valley, located in south-central Washington, USA, is home to the reservation of the Confederated Tribes and Bands of the Yakama Nation, and many communities, including Latinx farmworker families. Agriculture is the main economic driver in this region. Episodic poor air quality impacts the valley, with multiple sources of particulate matter, including residential wood smoke, smoke from wildfires, agricultural biomass burning, other agricultural emissions, and backyard burning. In 2015, Yakima was listed as one of only two communities in Washington in danger of exceeding the US Environmental Protection Agency (EPA) 24-hour standard for fine particulate matter [19]. Due to stagnant atmospheric conditions common in the wintertime in Yakima Valley, the county (for its jurisdiction) and the EPA (for the Yakama Reservation) issue bans on outdoor burning and restrictions on woodstove use.

University of Washington (UW) partnered with the Yakama Nation Environmental Management Program (EMP) and local institutions, Heritage University and White Swan High School, to develop CEnR on wood smoke, called Next Generation Sensors and Scientists (NextGenSS). NextGenSS had two main activities: to develop air quality research relationships with Yakama Nation, and to train Heritage University students to mentor White Swan High School students to design and implement their own research projects regarding air quality using low-cost air sensors. The NextGenSS team worked with a Project Advisory Committee (PAC) consisting of community partners. Heritage University and White Swan High School are located in rural towns on the Yakama Nation Reservation, about 2.5 hours’ drive from UW in Seattle.

**Figure 1:**
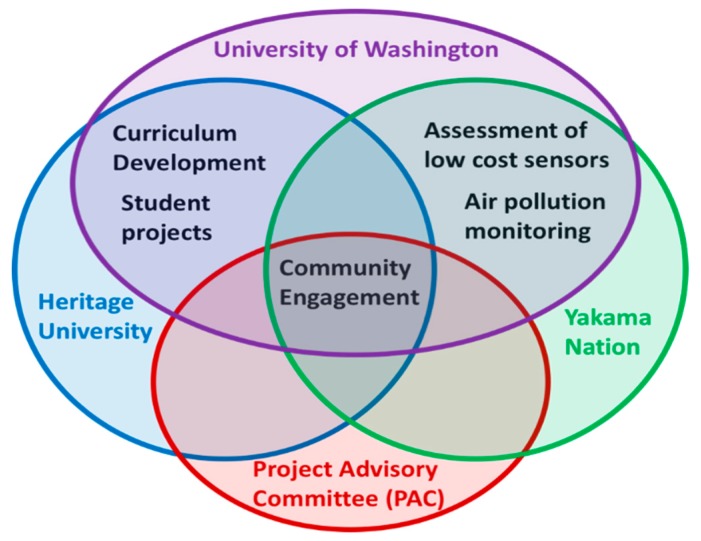
Academic and community partners involved in different aspects of NextGenSS.

The UW team was comprised of faculty, staff, and students. UW partnered with air quality staff from the Yakama Nation EMP, faculty and students from Heritage University, and the PAC. UW-Seattle is a state university with over 45,000 students. Heritage University is an independent, non-tribal university and designated Hispanic Serving Institution located on the Yakama Nation Reservation, and serves fewer than 1000 students. White Swan high school serves fewer than 250 students. Yakama Nation EMP oversees a variety of environmental quality needs on the reservation, including air, water, solid waste, hazardous materials, soil, wildlife, culture, and health. The goal of the Air Quality Section is to protect health, resources, and culture of Yakama Nation. The PAC was comprised of community leaders and agency representatives from Yakama Nation, a local Spanish language radio station, Indian Health Service, a tribal health focused non-profit, and the Mt. Adams school district (which contains White Swan High School). Figure 1 shows which partners worked together on different aspects of NextGenSS.

While the academic team had a history of research engagement with the Latinx agricultural community in the Yakima Valley, this project developed new connections between UW and Yakama Nation, and the interviewer joined at the beginning of this new engagement. The interviewer is a white, non-Native, non-Latinx student, who was part of the UW research team and supported the project’s programmatic and research objectives.

## 2. Materials and Methods

**Figure 2:**
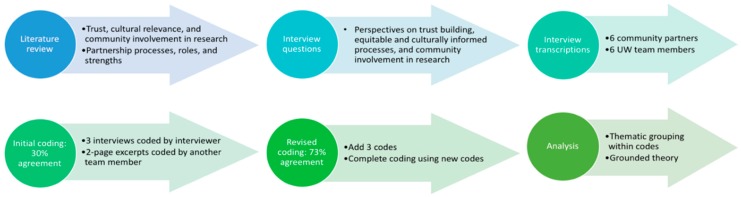
Outline of interview development and analysis.

Interview development and analysis steps are outlined in Figure 2. Interview questions were formulated from a literature review examining the relationships between trust, cultural relevance, and community involvement in research with partnership processes, roles, and strengths. Interview questions are included in Supplementary Materials (S1). Twelve semi-structured interviews were conducted with individuals from the community–academic partnership: 5 PAC members (out of 7), 6 UW research team members (out of 6), and 1 academic partner from Heritage University (out of 2). Because Heritage University is small and has strong ties in the surrounding rural Yakima Valley community, for the purposes of this analysis this academic partner was categorized as a community partner. Interviews lasted from 22 to 90 minutes, were either in-person or over the phone, and were recorded with participants’ permission. All interviews occurred between September and December 2017. Prior to any interviews, this study was submitted to the UW IRB (STUDY00003057) and US EPA Human Subjects Regulations (HSR-000925) review and was determined by both to be exempt on the 28th August, 2017 and 12th September, 2017, respectively. All subjects gave their informed consent for inclusion before they participated in the study.

Each interview was transcribed using a combination of audio-to-text and typing using Wreally Studios’ Transcribe software [20]. After transcribing all of the interviews, members of the research team for this study looked at findings and discussed ways to select codes based on major themes that emerged. This guided the selection of 31 codes, described in Supplementary Materials (S2). These codes each related to different aspects of relationship building, research processes, and community qualities. Dedoose software was used to apply codes to interview excerpts [21]. Following the coding of 3 interviews, another team member coded 2-page selections from each of the 3 interviews. These codes were in agreement about 30% of the time. In total, 62% of the discrepancies came from differing perspectives on the scope of “Trust Built” and “Trust Eroded”. It was agreed that an additional code related to communication was also needed. In response to this, 3 codes were added, described in Supplementary Materials (S2). This raised agreement in coding to 73%.

Once the code selections were complete, a close reading of each interview was performed, and codes were applied as themes appeared. Within each code, excerpts were grouped thematically, and emerging patterns were noted. For analysis, a grounded-theory approach was used, which is based on open-mindedness [22] and the lack of preconceived notions [23]. This approach does not test hypotheses; it instead generates theories from the data to explain patterns that have emerged [23].

## 3. Results

**Figure 3:**
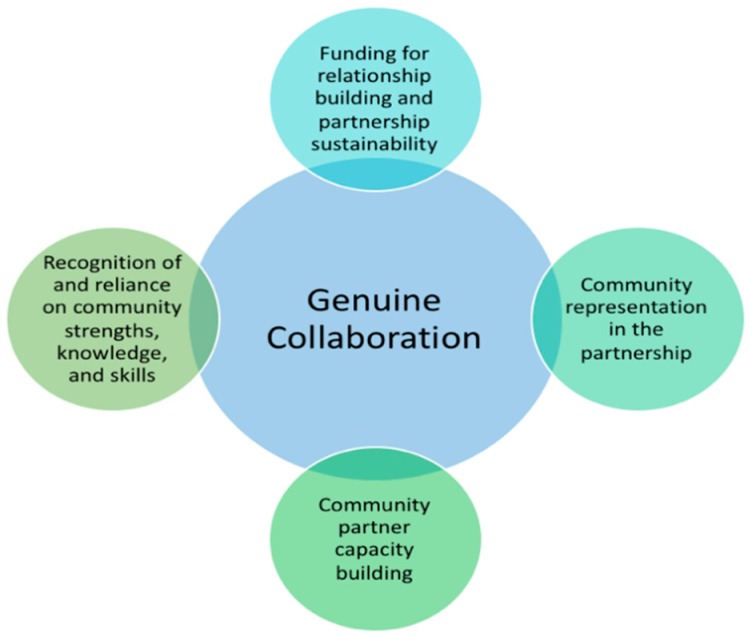
Major themes identified as elements of genuine collaboration.

Four themes were identified as important elements of genuine collaboration (Figure 3): (1) funding for relationship building and partnership sustainability, (2) community representation in the partnership, (3) recognition of community strengths (including relying on community partner knowledge and skills), and (4) community partner capacity building. 

Each of these themes is described below, including select supporting quotations. To respect confidentiality and privacy of respondents, each person is referred to as either a community partner or an academic partner, as categorized in the methods section. Each respondent approved of the use of their quotations.

### 3.1. Theme 1: Funding and Sustainability

The limited nature of individual grants and grant-specific budgetary restrictions often challenge collaboration development, relationship building, and project sustainability. Academic partners pointed out that the grant, while community focused, did not fund relationship building, despite its centrality to project success. An academic partner commented on the time and resources required for relationship building: “I am learning that what it takes to really begin is all of this foundation-building work outside of the project’s scientific activities”. This partner continued: “This is a struggle that we have on our side, learning and recognizing what it takes and the resource commitment to this part of it so that everything else, in terms of the project activities, can happen. Funding agencies and grant mechanisms don’t address this necessarily”.

Both academic and community partners expressed that, for community partners, academic resources were an important prospective benefit but participation in the project was only potentially useful. An academic partner suggested that perhaps for community partners: “it’s still probably a little bit of a gamble for them in a daily way when they’ve got so many other things in a resource poor environment to deal with. Is it really worth squeezing to deal with this stuff that maybe will pay off, but they don’t really know yet?” A community partner spoke directly to this issue: “The problem with it is that I feel there’s kind of a power struggle…. Part of my issue is that we’re having to take on additional tasks for this project. When I have staff that are already at full capacity for their positions, and then having to take on additional tasks for this project specifically, it’s going to be a bit of a juggling act, so that frustrates me, but at the end of the day I would like to think that, yes, this will be mutually beneficial.” Another community partner expressed that they were pleased with how funding was allotted: “I think it’s really good for the students to get appropriately compensated. I feel I am. There was money to buy some supplies at the end because we had extra so that was really good”.

Both academic and community partners discussed the short time-frame of research grants and how that impacts sustainability. A community partner explained: “These grants do have a lifetime and an end, but we’ll still be here and we’ll still be working with these kids and we’ll still be continuing on [the program]…. For us we will have to continue this work because we can’t stop, because there’s an expectation that no matter what, we don’t just leave, we stay…. Funded or not we find a way to keep going. For us, whenever we take on a commitment like this, it’s a much longer commitment than people realize”. An academic partner asked: “What happens at the end of this research project when the funding ends? Is anyone talking about that? There are very specific constraints that affect everyone on this project, academics and non-academics, the students, etc., our PAC members. To have some expectations of what would happen a year and a half from now would be helpful potentially. That sort of gets around the most egregious criticisms of community-engaged research, is that the academics helicopter in, drop in, parachute in, or whatever, and then fly out afterwards.”

### 3.2. Theme 2: Community Representation

Community representation was related to cultural diversity, dynamics between different organizations and agencies present in the community, and individual versus organizational voices. Academic and community partners noted that the community is diverse, and it was important to have representation on the project from different parts of the community, especially including both Latinx and Native community members. A community partner noted: “A big chunk of our population is our Hispanic families as well… There’s some dynamics there to think about. And I think the best way is to have those partners in the research, involved in the research”.

One community partner raised the issue that even if a partner organization is contacted, that does not mean that the necessary people at the organization were included properly. This partner said: “I think that the fact that the [organization] wasn’t involved in the very beginning of the process, I feel like we’re all kind of playing catch up” and “I wish we would have been involved from the very beginning.” Academic and community partners also acknowledged that initially the partnership did not adequately explore existing relationships and dynamics between organizations and agencies relevant to air quality. This resulted in the project partners unintentionally disrespecting tribal sovereignty. The same community partner explained: “You have to be really careful in how you build those partnerships outside the tribe. And you may be thinking that you’re doing a benefit for the tribe, when really you’re just stirring up old wounds.” Reflecting on community dynamics in general, an academic partner asked: “Did we do enough homework on what’s already going on and where people are convening?”

Additionally, partners raised concerns that most of the people involved in the project are representing organizations or agencies, but it is important to also have people involved who are just interested individuals. A community partner noted: “We’re all community partners, but I notice we’re all representing a different entity rather than getting people from the community itself. Maybe identify one or two people from the community. As community members we bring the personal perspective, but we also bring the perspective of the work we do at our organizations…if you bring in a community member it’ll be more about how their day-to-day life is. I think if you bring in a couple members who are really community, like a house-mom or an elder or a young person to be part of it, it would be helpful in understanding the community”. An academic partner echoed this suggestion: “I’m not sure if they have any active community members that aren’t part of the organizations that we’ve reached out to, like if one or two of them could join. Somebody that just cares about air quality”.

An academic partner commented on how the people collaborating on the partnership shape how the project reaches the community in general: “You’re trusting those people to represent a larger group…by the time you go to the larger group, there has been some crafting of what you’re saying, and it is through the lens of that smaller group of people that you’ve identified.”

### 3.3. Theme 3: Recognition of Community Strengths

Community and academic partners noted that focusing on community strengths, including local knowledge, networks, previous related work, and cultural awareness, is necessary to support a genuine relationship. They commented that community partner strengths may be especially valuable in air quality program implementation, community engagement, and curriculum development. A community partner raised the idea that early communication, before decisions have been made, is highly important: “We’re being heard fairly well. I just wish that there was more communication as far as when different ideas come up that we would be reached out to, to say ’Hey what do you guys think of this? Have you done this? Do you have any information available?’ rather than putting things in order and trying to get stuff set up and then coming in at the last minute and saying, ’oh yeah what do you guys think?’ when we could be more beneficial at the very upfront.” Another community partner expanded on this idea: “One way is to say ’you’re the experts in the implementation and how this would be done. How can you help us in looking at the factors and the variables of what this program might look at?’…. It needs to be a genuine engagement of something that that tribe or that community has to offer in the process, otherwise they’re going to see through it at some point”. This partner continued to note that projects or programs built with the community’s knowledge are much more likely to be successful: “Those are the pieces that need to be built up completely in tandem, 100% with the research on evidence-based practices”. As this project did rely on community partners and community strengths for implementation, a shift in power was recognized by an academic partner: “Sometimes I feel like I have less power over this thing, because it’s really kind of up to a lot of these other people to be involved and do things to make it all happen.” 

Community and academic partners conveyed the importance of multi-directional learning and teaching. This involves academic partners learning from community partners, instead of the more typical approach where academic partners are considered “the experts” who teach community partners. One community partner said: “I think that we all are lifetime learners and so I think that there’s a lot that myself and my staff can learn from University of Washington, but I also think that UW folks can learn a lot from us…living the life and traditions that we live, and help you gain an understanding of our tribe and our culture. I think we have a lot to share between each other”. This partner also described the community strength of traditional knowledge, and the importance of collaboration including different kinds of knowledge: “TEK is so important - traditional ecological knowledge. You know, the Yakama people have lived on the planet since time immemorial, and we’ve lived here harmoniously with the environment, so I think it’s important to look back on those things and take those lessons…. TEK absolutely needs to be hand in hand with modern science”.

### 3.4. Theme 4: Community Partner Capacity Building

Community partners expressed appreciation for academics’ assistance in data interpretation and general support. An important note voiced by a community partner was that building community partner capacity must be genuine. This community partner noted that research money often goes to academic institutions working with community partners. This partner said: “I don’t want to call it token, but there’s a certain degree of ‘the more we have you involved the more likely we are to get this grant’ which is great but it’s sort of holding the community’s capacity in as a feature versus as the driving component.” Community partners expressed that genuinely building community partner capacity may lead to more equitable benefits from the research between the two groups. One community partner mentioned a capacity building need in air sensor knowledge that would promote richer participation: “In terms of the instruments, I don’t know what’s available, I don’t know what the actions are. Since that’s not my expertise I’m not sure I feel comfortable saying yes or no to some of these things”. 

Academic and community partners both discussed goals for building community partner capacity, but they had slightly different areas of focus. Academic partners expressed desires to build community capacity to “identify some resources that are beneficial”, “write a grant…drive their own interests”, and do “outreach and engagement.” Community partners discussed “technical expertise”, “roles of research and investigations”, and “public health”. An academic partner also voiced that, without dialogue, community partners may not even know what is available to them through the project. This partner suggested: “We can just ask them what are some other things that you need, what would you like, maybe there’s some other things we can provide to you. Just having an open conversation acknowledging the fact that we might be able to provide you more than what you’re asking for”. A community partner emphasized that community partner roles should reflect capacity building needs: “If formal roles were genuine, and emphasized capacity-building, then you would probably be getting closer to building actual relationships”.

## 4. Discussion

### 4.1. Fund Relationship Building and Community Partners’ Involvement

Building relationships is an essential part of the CEnR process, particularly with Native communities [24]. Community and academic partner comments suggest that if community partners are not being compensated for their time spent relationship building, their participation is risky in the sense that their time and energy investment may not result in a benefit to them or their community. Additionally, a power dynamic emerges from community partners not being compensated for their time (on relationship building and/or on project activities) and only potentially standing to benefit from the relationship. While appropriate compensation for community partners is essential, it does not eliminate this power dynamic because decision-making power about money still lies with the academic partners. This power dynamic may be an obstacle to a genuinely collaborative partnership.

Funding for relationship building on the academic side is also important because it facilitates spending the necessary time and resources to build deeper relationships. Funding and support for relationship building outside of the scope of a grant allows for relationship building to occur prior to starting research. Collaboration outside of a research project also facilitates mutual respect, mutual trust, and multi-directional learning. Academic and community partners should discuss the range of funding options possible, and avoid academic partners making assumptions about community partners’ funding preferences.

This emphasis on funding for relationship building is mirrored in other CEnR partnerships and evaluations. Others have found it beneficial to financially compensate members of community boards [25], use funds to support partnership infrastructure [26], financially compensate community partners’ time spent on the project [27], and hire community partners and members as salaried staff written into the project budget [28]. Wolff and Maurana found that hiring community members and writing community partners into the budget was important to communities participating in CEnR [29]. A participatory collaboration in Detroit shared in one of their five main recommendations that community partners need to be compensated for their time and expertise [16].

Funding agencies could play a role by emphasizing the value and necessity of relationship building, and funding the time and resources required for both community and academic partners [7,30]. A review of health-related participatory research with Native communities found that meaningful relationship building was challenged by time and funding allotted in grants [31]. The National Institutes of Health-funded Center for American Indian Resilience and the Southwest Health Equity Research Collaborative has a unique funding mechanism to financially support relationship building for both academic and community partners [32]. This mechanism allows time for trust building and making sound decisions about whether or not to move forward with the collaboration, for example to write a grant proposal together [32].

### 4.2. Plan for Sustainability

Short research time-frames contribute to the occurrence of “helicopter research”, where academics merely collect data from a community and fail to report results or otherwise involve the community in any meaningful way [12,24]. This style of research can cause communities to rightfully be wary of research and make it more difficult to involve community partners [12,24]. Academic partners shared a desire to avoid “helicopter research”. Community partner comments suggest that limited time on research grants also creates barriers to having an equitable partnership; community partners often need to continue related or grant-initiated work past the end of the grant, while academics typically work on the day-to-day progression and maintenance of a project within the grant time-frame, with paper writing often remaining afterwards. This places extra pressure on the community partners. 

Again, these findings are echoed by previous studies. A partnership evaluation found that commitment to long-term sustainability was one of five main recommendations [15]. Commitment to continued work past the end of the study was also one of the ten principles developed to guide health research with Indigenous Australian communities [33]. Communities participating in CEnR expressed a desire for sustainability in research interventions [26], in the partnership itself [27,34], and in impacts arising from the research [27,30]. Sustainability relies on continued funding, academic dedication to enduring support for the partnership, and community partners’ involvement and power in the partnership [29].

Long-term commitment may support genuine collaboration because it demonstrates a shared interest in the well-being of the community beyond the scope of the current study or a single funding opportunity. Additionally, long term partnerships may facilitate greater opportunities for multi-directional learning and teaching between academic and community partners.

### 4.3. Strive for a Representative Partnership 

Superficial collaboration might involve seeking out a community partner and then “checking the box” of having a community voice present. Authentic collaboration may demonstrate a commitment to representing the larger community more accurately, which means learning about different perspectives in the community and how they are represented. Then people and organizations can be invited to the partnership accordingly, while being cognizant of the voices and perspectives not at the table. In the partnership studied here, being intentional about having members from both Latinx and Native communities was important, as well as being mindful when contacting organizations that not all individuals at the organization have necessarily been contacted. Community representation is also complicated by organizational versus individual perspectives, and it is valuable to include community partners who do not represent an organization. Tribal sovereignty and dynamics between different organizations and agencies also play a role in who is part of the partnership.

Some of these findings are reflected in guidance on research partnerships and evaluations. Regarding diversity within the community and dynamics between groups, Duran et al. caution that academic researchers need to be aware of the fact that a single community entity contains a multitude of perspectives and opinions [35]. Smaller groups within the community may have different stances on an issue [35]. Ross et al. note that community leaders may or may not accurately represent the larger community [34]. Recognizing diversity within Indigenous Australian communities was another one of the ten principles developed to guide health research with Indigenous Australian communities [33]. A review of community-institutional partnerships found that partners need to understand the diversity of the community and how the community is represented to inform who should join the partnership [30]. This might include seeking to meet and build rapport with a wide range of individuals and entities within a community. Regarding individual versus organizational perspectives, guidance on partnerships suggest that it is helpful to have formal leaders, informal leaders or activists, and regular people involved in the project [36,37].

Two issues raised here were not generally reflected in the literature: differentiating between making contact with an organization and meaningfully connecting with an organization, and ways that tribal sovereignty may impact who is part of the partnership. Federally recognized tribes are sovereign nations that maintain a government-to-government relationship with the United States federal government. Therefore, it may be inappropriate to involve agencies from lower levels of government in the research. Academic partner cautiousness, humility, and respect around this issue is necessary. Further guidance on both of these issues in research partnerships would be valuable.

### 4.4. Rely on Community Strengths for Project Success

Community and academic partner comments suggest that asking community partners for their help before the grant is even written is a form of strengths recognition because it makes it more likely that the project will lean on community partner strengths. Relying on the capabilities and wisdom of community partners also shifts the power dynamic. Community strengths related to implementation may help the project be more beneficial to the community, and also may make it more sustainable. These strengths should inform how the research is conducted. When the partnership and project success rely on the knowledge, expertise, and skills of community partners, that may support a more genuine collaboration as different parties involved take ownership of the project together. Relying on community strengths also facilitates mutual teaching and learning between academic and community partners.

These ideas are noted in previous studies and guidance on partnerships. Duran et al. describe several key elements in establishing and nurturing community partnerships [35]. These include emphasizing the community’s assets, and conducting a strengths assessment in addition to a needs assessment [35]. Koné et al. note that sharing power and recognizing what the community has to offer are vital to CEnR [37]. Christopher et al. suggest that when academic partners express that the community partners are experts in their fields and in their community, community partners are more likely to participate [11]. Communities participating in CEnR expressed a desire for greater involvement and responsibilities aligning with their knowledge and skills [37], and to be acknowledged as experts [28,29]. Collaboration is more likely when there is recognition of community strengths [26,27,30] and when community partners are involved in all stages of research. This helps ensure that the project relies on community knowledge and skills [7,26]. Collaboration with tribes specifically is strengthened by respecting cultural and traditional knowledge and indigenous methodologies [7]. The Center for American Indian Resilience and the Southwest Health Equity Research Collaborative funding mechanism mentioned above resulted in partners discussing innovative strategies together [32]. This type of discussion implies that partner expertise is valued and respected, as opinions on new approaches are sought.

### 4.5. Build Community Partner Capacity According to Community Priorities

Perspectives provided in these interviews suggest that academic partners need to be clear about what they might be able to offer, even if community partners do not ask. A dialogue that moves the partnership away from negotiation or transaction style interactions and towards a mutually supportive and equitable collaboration is important. Research benefits may be more equitably distributed when research activities and partner roles are driven by community partner capacity building priorities. When community roles are based on existing strengths and capacity building priorities, and have power, the partnership is strengthened by avoiding tokenizing community partners, an idea also mentioned by Butterfoss [12]. Building capacity also helps equalize decision making power because all of the parties become more informed about the decisions.

Building community partner capacity is featured in several evaluations and partnership guidance. Community capacity building was one of seven elements in the partnership model of Wilson et al. [9]. Another one of the ten principles developed to guide health research with Indigenous Australian communities is that capacity building is a main emphasis of the partnership, and they note this emphasis should be backed up financially [33]. Jamieson et al. note that capacity building may be accomplished through hiring indigenous community partners and encouraging their career development [33]. Importantly, they further note that having indigenous partners on staff facilitates non-indigenous staff learning [33]. This idea ties back to the complementary nature of relying on community strengths and mutual teaching and learning. One distinction made in our analysis is that community partner capacity building should be based on community priorities—community and academic partners may have different ideas of which skills should be prioritized.

### 4.6. Limitations and Strengths

One limitation in this study is that the interviewer was part of the UW research team, and their views were biased. Using grounded theory helped to reduce potential bias, as it encourages the interviewer to abandon preconceived notions and engage in self-reflection. The interviewer strove to remain aware of their positionality as a white, urban student and member of the UW team. At the same time, conducting interview coding and analysis manually introduced more potential for bias. Comparing coding with another coder helped reduce this bias, though disagreement on code definitions contributed to poor initial validity. With the clarification and addition of new codes, validity increased. Another important limitation is that community partner interviewees may not have felt comfortable sharing openly with an academic partner. This may impact how well the interview results reflected partners’ ideas. This limitation may have been mitigated by the open-ended, neutral nature of the interview questions, and the relatively low power attributed to students compared to other academic partners. 

Despite those limitations, nearly all partners freely shared vulnerabilities and concerns, which suggests some level of comfort during the interviews. Another strength of this study is that the community–academic partnership was well-represented, with 12 out of 15 members (at the time of the study) participating. This study was conducted early in the process of establishing relationships, which maximized its potential impact to improve the partnership. This study also addresses a wide range of relationships, as this partnership was multicultural, urban-rural, included a tribal government program, and included both a large, urban, public university and a small, rural, community-based university.

### 4.7. Implications

**Figure 4:**
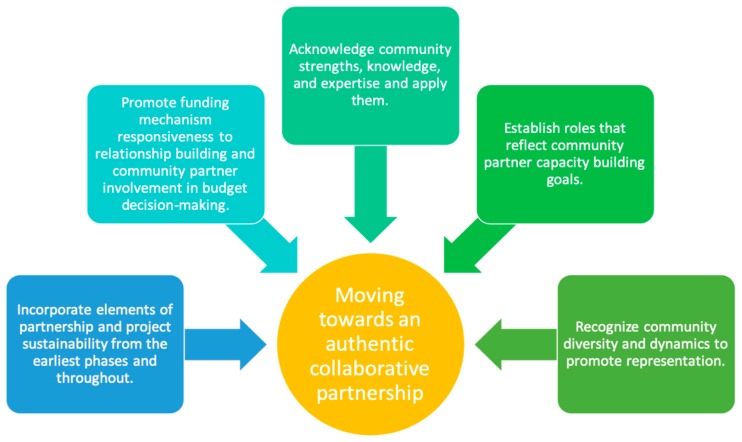
Guidance for an authentic collaborative partnership based on partner interviews.

Based on the results and discussion of community and academic partner interviews, actions that support genuine collaboration include (1) incorporating elements of partnership and project sustainability from the earliest phases and throughout, (2) promoting funding mechanism responsiveness to relationship building and community partner involvement in budget decision-making, (3) acknowledging community strengths, knowledge, and expertise and applying them, (4) establishing roles that reflect community partner capacity building goals, and (5) recognizing community diversity and dynamics to promote representation (Figure 4). 

Since these interviews took place in 2017, the three-year funding cycle for NextGenSS has concluded. We have sought to support sustainability by moving from Heritage University and White Swan High School partners borrowing UW air monitoring instruments to community partner ownership of 20 air monitors to be used in future high school research projects. Relationship building was incorporated into other project deliverables, such as PAC meetings, air monitoring research, and community engagement activities. Community knowledge drove the implementation of the air quality curriculum and planning of community engagement activities. Heritage University students expressed interest in building capacity in certain research topics and skills, and UW researchers responded with additional support in those areas. Community partners represented the diversity of the community as a whole in some key components and not others. Community partners provided input on community organizations and agencies that were more or less likely to foster the project goals and partnership.

The focus of the partnership has changed to emphasize the collaboration between Yakama Nation EMP and UW researchers. In developing this more specific partnership, we are striving to apply the lessons learned from this analysis. For example, we wrote a successful grant proposal together that grew from EMP staff interests and priorities, and compensation for EMP involvement in the project was written into the budget. EMP staff knowledge and expertise drive research design and data interpretation. EMP staff have expressed interest in building capacity in specific areas, and roles have shifted to facilitate skill building in those areas. EMP staff and UW researchers continually learn from each other throughout the process.

## 5. Conclusions

The themes highlighted in this qualitative study enrich lessons learned in prior CEnR evaluations and reflections, and provide guidance for fostering sustained success in academic-community partnerships. These lessons may be especially useful for tribal, urban-rural, and multicultural research partnerships. These lessons are also valuable for foundation and government funding agencies that promote community engaged research.

## Supplementary Materials

The following are available online at https://www.mdpi.com/1660-4601/16/24/5132/s1, S1: Interview questions, S2: Code definitions.
