# Supplementary material for: Guidance for Genuine Collaboration: Insights from Academic, Tribal, and Community Partner Interviews on a New Research Partnership
Source: Int J Environ Res Public Health. 2019 Dec 16;16(24):5132. doi: 10.3390/ijerph16245132 (PMC6950304; doi:10.3390/ijerph16245132)
Supplement: Supplementary file 1 [file ijerph-16-05132-s001.zip › Supplementary materials/S1 Interview questions.docx]

**Supplementary Materials**

S1: Interview questions

1. What is your goal in participating in this group?

- *Probe*: Is your participation in this team beneficial to you?
- *Probe*: What is your vision for the research?

1. How do you feel that your voice was heard in defining the issues?

1. How do you feel that your expertise is currently acknowledged by the team?

- *Probe*: What opportunities would you want to share your expertise?

1. How do you think that this research will benefit the public health of this community?

- *Probe*: Any parts of the community in particular?

1. What does trust look like to you?
2. Thinking of trust in the research partnership, what do you think would increase trust?

- *Probe*: Do you have recommendations for particular partners in the research partnership?

It is thought that more formal roles with explicit expectations and specific tasks can help to build trust among research partners.

1. How do you think this might impact team trust?

- *Probe*: How do you think this might impact your involvement in the team?

(Ask only if there is extra time)

Do you recommend any decision-making processes that may be helpful in building trust among the partners?

- *Probe*: Do you think that it would be helpful to have a more formalized process in place for addressing conflict when it arises?

In other community-engaged research projects, academic and community partners have suggested that for a trusting partnership, academics should reflect on personal and institutional histories, and the historical context of research and research abuses.

1. How do you think this type of reflection might help build team trust?

- *Probe*: Do you think this is something we should do altogether, or just the academic partners on their own?

1. Given differences in resources and power between UW and PAC members, what are some ways you think our team can build equitable and respectful relationships?
2. How could our partnership as a whole best gain an understanding of the cultural histories of the communities at the center of this research project?

When research partners are more present in the community, it may help to facilitate community involvement in the research process and in the health actions that follow.

1. What recommendations do you have about partners being more present in the community?
2. How could our partnership as a whole best demonstrate respect for this community’s cultural beliefs, practices, and tribal sovereignty?

- *Probe*: Can you think of any ways to demonstrate this respect as part of the research process?

1. How could this research be better informed by the cultures of the communities we’re working with?

- *Probe*: How could the air pollution curriculum for the students be more culturally relevant?

One way to describe traditional knowledge is information passed down through generations based on observation, use, and closeness to an area or ecosystem. Traditional knowledge may be passed down through oral tradition.

1. What roles do you think traditional knowledge has in our research process?

1. How can we improve participation in the project by all partners, including meeting attendance and sharing ideas for the project?

- *Probe*: Would you feel more connected to the group if we built in time for socializing as part of the meetings?
- *Probe*: What kind of contact and communication between meetings would help you feel more involved?
