# Supplementary material for: Guidance for Genuine Collaboration: Insights from Academic, Tribal, and Community Partner Interviews on a New Research Partnership
Source: Int J Environ Res Public Health. 2019 Dec 16;16(24):5132. doi: 10.3390/ijerph16245132 (PMC6950304; doi:10.3390/ijerph16245132)
Supplement: Supplementary file 1 [file ijerph-16-05132-s001.zip › Supplementary materials/S2 Code definitions.docx]

**Supplementary Materials**

S2: Code definitions

| Code type | Code | Meaning |
| --- | --- | --- |
| Primary | **Trust Built** | Examples of trust being built in the partnership |
| Secondary | Being listened to | People expressing that they are being listened to |
| Secondary | Proxy trust and functional trust | Examples of specifically proxy trust (trust based on connections through another person or entity) and functional trust (trust being forced because of need for project to move forward) in the partnership |
| Primary | **Trust Eroded** | Examples of trust being eroded in the partnership |
| Secondary | (Lack of) Academic due diligence | Examples of lack of academic due diligence in choosing community partners, planning the project, etc. |
| Secondary | Divisions within community | Examples of evidence for divisions within the community, or between community partners |
| Primary | **Transparency** | References to the idea of transparency in research, including clarity of project roles and motivations, and updates |
| Primary | **Research** | References to issues related to research needs, grants, timelines |
| Secondary | Funding | References to funding including issues with grant rigidity |
| Secondary | Grant time lag | References to the long time period between applying for the grant and starting the project |
| Primary | **Sustainability** | References to the sustainability of the research – what happens to all of the components of this (including the partnership) after the 3 years of the grant is over? |
| Primary | **Community context** | Information about the community that is important to inform the way we do this project together, including cultural aspects, socioeconomic situation, relationships, and history. |
| Secondary | Concern about representation | References to how the project is represented; UW and HU’s reputations in the community |
| Secondary | Multiple perspectives | The community is made up of many different groups and cultures – references to the diversity of perspectives in the community |
| Secondary | Practical actions | References to the need for information on practical actions individuals in the community can take to better their health |
| Primary | **Community strengths** | References to community strengths |
| Secondary | Elders | Potential or actual roles of elders in the community or project |
| Secondary | Youth | Potential or actual roles of youth in the community or project |
| Primary | **Community motivations** | Motivations for community members to participate in the partnership (from community partner interviews) |
| Secondary | Learning | References to community members wanting to learn more (from community partner interviews) |
| Primary | **Tribal context** | Similar to Community context, but tribal specific. |
| Primary | **Academics clarity of intent** | Intentions of academics in participating in the partnership (from academic partner interviews) |
| Secondary | Academics learning | References to academics wanting to learn more (from academic partner interviews) |
| Secondary | Issue identification | How academics describe issue identification (from academic partner interviews) |
| Primary | **Community capacity building** | References to community capacity building |
| Primary | **Academic presence in community** | References to how academics should be present in the community |
| Primary | **Concrete suggestions/ideas** | This code is to keep track of concrete suggestions or ideas interviewees had for improving the partnership |
| Primary | **Visions** | This code includes answers to the question “What is your vision for this project?” |
| Primary | **Goals** | This code includes answers to the question “What is your goal in participating this project?” |
| Primary | **Definition of trust** | This code includes answers to the question “What does trust look like to you?” |
| Primary | **Formalization** | References to formal roles, processes, and agreements. |

Codes added:

| Code type | Code | Meaning |
| --- | --- | --- |
| Primary | **Trust Built – Potential or General** | Examples of ways that trust has been built within the community or between the community and outsiders in general, or ways that trust could be built |
| Primary | **Trust Eroded – Potential or General** | Examples of ways that trust has been eroded within the community or between the community and outsiders in general, or ways that trust could be eroded |
| Primary | **Dialogue** | References to frequency of communication and quality of communication |
